# Intracranial arterial calcification in patients with unruptured and ruptured intracranial aneurysms

**DOI:** 10.1007/s00330-024-10789-2

**Published:** 2024-05-28

**Authors:** Maarten J. Kamphuis, Laura T. van der Kamp, Edwin Lette, Gabriel J. E. Rinkel, Mervyn D. I. Vergouwen, Irene C. van der Schaaf, Pim A. de Jong, Ynte M. Ruigrok

**Affiliations:** 1 Department of Radiology, University Medical Center Utrecht, Utrecht University, Utrecht, The Netherlands; 2 Department of Neurology and Neurosurgery, UMC Utrecht Brain Center, University Medical Center Utrecht, Utrecht University, Utrecht, The Netherlands

**Keywords:** Brain, Intracranial aneurysm, Physiologic calcification, Subarachnoid hemorrhage, Tomography (x-ray computed)

## Abstract

**Objectives:**

Arterial calcification is thought to protect against rupture of intracranial aneurysms, but studies in a representative population of intracranial aneurysm patients have not yet been performed. The aim was to compare the prevalence of aneurysm wall calcification and intracranial carotid artery calcification (ICAC) between patients with an unruptured intracranial aneurysm (UIA) and a ruptured intracranial aneurysm (RIA).

**Materials and methods:**

We matched 150 consecutive UIA patients to 150 RIA patients on age and sex. Aneurysm wall calcification and ICAC were quantified on non-contrast enhanced computed tomography images with the modified Agatston score. We compared the prevalence of aneurysm wall calcification, ICAC, and severe ICAC (defined as a modified Agatston score in the fourth quartile) between UIA and RIA patients using univariate and multivariate conditional logistic regression models adjusted for aneurysm characteristics and cardiovascular risk factors.

**Results:**

Aneurysm wall calcification was more prevalent in UIA compared to RIA patients (OR 5.2, 95% CI: 2.0–13.8), which persisted after adjustment (OR 5.9, 95% CI: 1.7–20.2). ICAC prevalence did not differ between the two groups (crude OR 0.9, 95% CI: 0.5–1.8). Severe ICAC was more prevalent in UIA patients (OR 2.0, 95% CI: 1.1–3.6), but not after adjustment (OR 1.0, 95% CI: 0.5–2.3).

**Conclusions:**

Aneurysm wall calcification but not ICAC was more prevalent in UIAs than in RIAs, which corresponds to the hypothesis that calcification may protect against aneurysmal rupture. Aneurysm wall calcification should be further assessed as a predictor of aneurysm stability in prospective cohort studies.

**Clinical relevance statement:**

Calcification of the intracranial aneurysm wall was more prevalent in unruptured than ruptured intracranial aneurysms after adjustment for cardiovascular risk factors. Calcification may therefore protect the aneurysm against rupture, and aneurysm wall calcification is a candidate predictor of aneurysm stability.

**Key Points:**

- *Aneurysm wall calcification was more prevalent in patients with unruptured than ruptured aneurysms, while internal carotid artery calcification was similar*.
- *Aneurysm wall calcification but not internal carotid artery calcification is a candidate predictor of aneurysm stability*.
- *Cohort studies are needed to assess the predictive value of aneurysm wall calcification for aneurysm stability*.

**Graphical Abstract:**

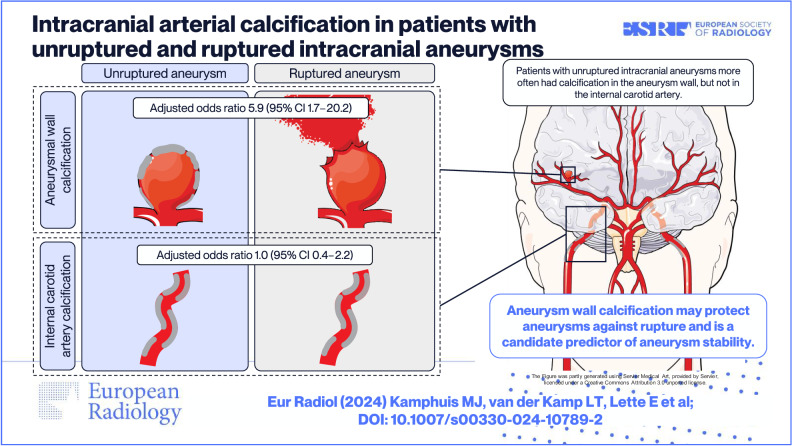

## Introduction

In patients with an unruptured intracranial aneurysm (UIA), the risk of rupture needs to be balanced against the risk of treatment complications. If the risk of rupture exceeds the risk of treatment complications, preventive treatment is usually advised and in other cases patients are monitored with follow-up imaging [1]. Identifying rupture-prone aneurysms remains challenging, and rupture risk prediction needs to be improved, which could be achieved with new imaging markers.

Calcification of the aneurysm wall may be a potential marker of aneurysm stability. Studies that compared calcification in unruptured intracranial aneurysms (UIAs) to ruptured intracranial aneurysms (RIAs) found that calcification in the aneurysm wall is more prevalent in UIAs compared to RIAs [2–4]. These studies were performed ex-vivo [4], in giant aneurysms [3], or in children [2], and it remains unknown whether the prevalence of calcification differs between patients with an UIA and those with a RIA in a representative adult population when assessed with clinical computed tomography (CT) scans.

Arterial calcification develops in two layers of the arterial wall: the tunica intima and the tunica media [5, 6]. Calcification in the tunica intima is predominantly atherosclerotic, and that in the tunica media nonatherosclerotic [6]. Atherosclerotic intimal calcification is thought to arise from an inflammatory response to smoking and hypertension [7–11], whereas nonatherosclerotic medial calcification is most likely caused by mechanical damage to elastic fibers or metabolic disease [7–13]. In an ex-vivo study, nonatherosclerotic calcification has been observed in both UIAs and RIAs, whereas atherosclerotic calcification was restricted to UIAs [4]. This distinction between the location (intimal or medial) could provide additional information on aneurysm instability. When using intracranial carotid artery calcification (ICAC) as a surrogate marker, atherosclerotic calcification in the intimal layer can be distinguished from nonatherosclerotic calcification in the medial layer on clinical CT scans [5, 14]. Since macroscopic calcification of aneurysms that can be detected by clinical CT is probably rare and macroscopic ICAC calcification is more common [14], this ICAC may also serve as a marker of aneurysm stability in the aneurysms that appear non-calcified on CT.

The aim of this study was to compare the prevalence of calcification in the wall of intracranial aneurysms between UIA and RIA patients on clinical CT scans, and to compare the prevalence and location of ICAC between UIA and RIA patients. We hypothesized that aneurysm wall calcification and ICAC, specifically in the intimal layer, is more prevalent in UIA compared to RIA patients.

## Materials and methods

### Study population

The institutional ethics committee of the UMC Utrecht waived the requirement for formal ethical assessment, since data from routine patient care were used (NedMec, 22-035/DB). We included patients from the University Medical Center (UMC) Utrecht, The Netherlands, a tertiary referral center for patients with intracranial aneurysms. We retrieved 150 consecutive saccular UIA patients whose UIA was diagnosed with CT angiography (CTA) between 2008 and 2021 from our institutional UIA database. These patients were matched based on age and sex in a 1:1 ratio with RIA patients diagnosed with aneurysmal subarachnoid hemorrhage on head CT. For age matching, a bandwidth of 3 years was used. We included patients ≥ 18 years old who were imaged with thin-slice head CT imaging, and excluded patients with multiple aneurysms (both in RIA and UIA patient group).

### Collection of patient and aneurysm characteristics

All patient and aneurysm characteristics were collected retrospectively from medical records at the time of aneurysm diagnosis. Patients were recorded as having hypertension, hyperlipidemia, or diabetes mellitus if it was either diagnosed by a physician or if the patient was taking medication prescribed for these conditions, regardless of whether these conditions were controlled by medication. One standard alcoholic drink was assumed to contain 10 g of alcohol, and alcohol intake was considered excessive at > 30 g per day. Smokers were categorized into current, former, or never smokers, with former smokers being defined as persons who stopped smoking at least 3 months before baseline. Aneurysm location and size were recorded from unstructured radiology reports. Aneurysm location was categorized into anterior cerebral and communicating artery, internal carotid and posterior communicating artery, middle cerebral artery, or posterior cerebral and vertebrobasilar arteries. Aneurysm size was specified in 281 of 300 radiology reports (94%). In the remaining 6% aneurysm size was measured by M.J.K. (> 2 years of experience in intracranial aneurysm measurements), L.v.d.K. (> 3 years of experience), or M.D.I.V. (> 15 years of experience) using an electronic caliper on multiplanar reconstructions of the aneurysm.

### Calcification measurements

Aneurysm wall calcification and ICAC were quantified using IntelliSpace Portal HeartBeat CS mode (Philips Healthcare). We quantified calcification in the aneurysm wall using the modified Agatston score by drawing regions of interest around calcification in the aneurysm wall on non-contrast enhanced CT slices and used CT angiography images to localize the aneurysm. Regions of interest were drawn by M.J.K. Blinding for aneurysm rupture status was not feasible since subarachnoid hemorrhage was often visible on CT in RIA patients. As proposed by Agatston for the coronaries [15], the highest attenuation value in Hounsfield units was determined per region of interest, and was assigned a density score. Density scores were weighted by multiplying with the surface area of the region of interest. The threshold for calcification lesions was set at 130 Hounsfield units [15].

**Fig. 1:**
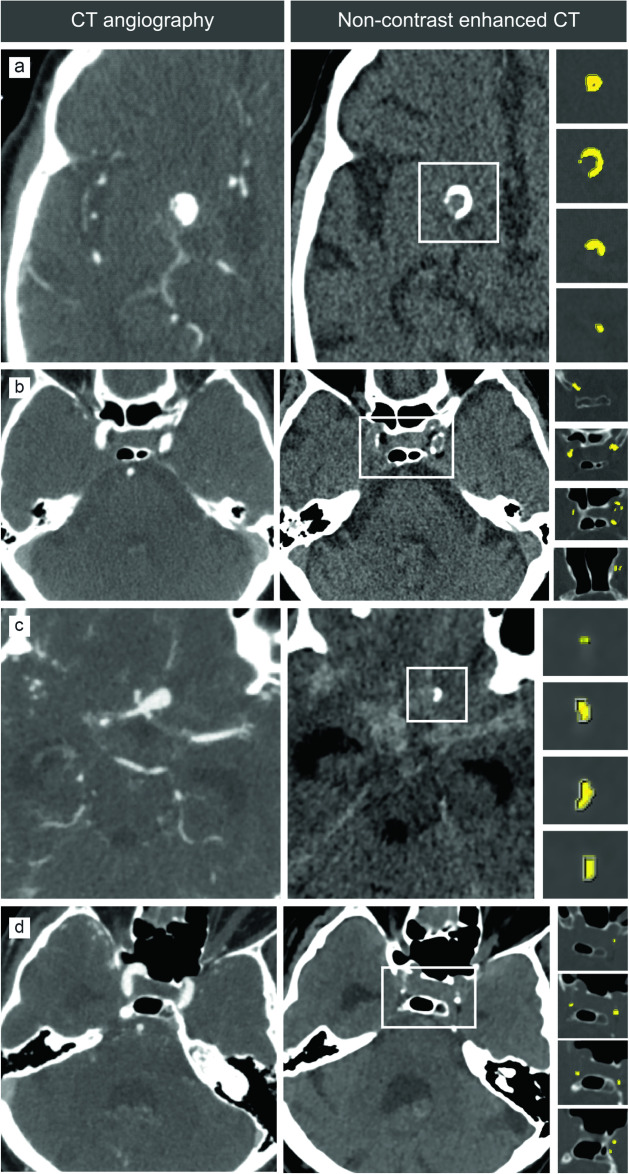
Calcification of the aneurysm wall and internal carotid arteries in representative unruptured and ruptured intracranial aneurysms. Yellow areas mark regions of interest, from cranial (top) to caudal. **a** CT angiography shows an unruptured 8-mm aneurysm of the right internal carotid artery tip, and non-contrast enhanced CT shows the calcified aneurysm wall (rectangle). **b** CT angiography and non-contrast enhanced CT show bilateral calcifications in the internal carotid arteries (rectangle). **c** Ruptured 10-mm aneurysm of the anterior communicating artery with a focal calcification in the aneurysm wall (rectangle). **d** Minimal calcifications along the course of the internal carotid arteries (rectangle)

We quantified ICAC with the modified Agatston score as described previously [14, 15]. Briefly, we drew regions of interest on non-contrast enhanced CT slices bilaterally along the course of the intracranial internal carotid artery from the petrous internal carotid artery segment to the carotid artery tip (Fig. 1). Regions of interest were drawn by E.L. or M.J.K., and cross-checked by a radiologist with > 10 years’ experience (P.A.d.J.).

ICAC location (intimal or medial) was determined using a previously developed scoring system that assigns points to three ICAC characteristics: circularity (absent, dots, < 90 degrees, 90–270 degrees, or 270–360 degrees), thickness (absent, thick ≥ 1.5 mm, or thin < 1.5 mm) and morphology (indistinguishable, irregular/patchy, continuous) [5]. ICAC located in the intimal layer tends to be thick, irregular, and non-circular, whereas ICAC in the medial layers tends to be thin, continuous, and circular [5]. Scores were assigned by a radiologist (P.A.d.J.). Scores < 7 were classified as intimal ICAC; scores ≥ 7 as medial ICAC. If ICAC was absent or the morphology was indistinguishable ICAC was classified into a single category ‘absent/indistinguishable’.

### Statistical analysis

We assessed aneurysm wall calcification as present or absent. Since modified Agatston scores were not normally distributed, a log transformation was applied, and to all non-transformed values 1 was added to be able to handle scores of 0 in our analyses. We divided ICAC modified Agatston scores into quartiles, and dichotomized ICAC severity into a severe group (fourth quartile) and a non-severe or absent group (first to third quartile). We compared ICAC prevalence, location and aneurysm wall calcification prevalence in UIA patients to RIA patients using conditional logistic regression models with rupture status as the dependent variable and controlled for the matching factors by including age and sex in the models [16]. A squared-transformation was applied to age. In model 1 we did not adjust for confounders, in model 2 we adjusted for aneurysm size and location, and in model 3 we additionally adjusted for history of stroke or transient ischemic attack (TIA), hypertension, hyperlipidemia, diabetes mellitus, smoking status, and alcohol intake. Aneurysm size was log-transformed and treated as a continuous variable; other variables were categorical. For aneurysm location, the middle cerebral artery was used as the reference; for smoking, the reference was never smokers. Five variables had missing values: hypertension (1 patient, 0.3%), hyperlipidemia (1 patient), diabetes mellitus (1 patient), alcohol intake (49 patients, 16%), and smoking status (22 patients, 7%). Data were missing randomly and were imputed with multiple imputation. Statistical analyses were performed with R statistical software version 4.2.1.

### Power calculation

A power calculation was performed for the crude association between internal carotid artery calcification and aneurysm rupture in the age and sex-matched cases and controls. Assuming an alpha of 5%, a power of 80%, and an expected odds ratio of 0.7 based on a previous histological study [4], at least 94 patients were needed per group. Since we used ICAC as a surrogate for aneurysm wall calcification in addition to measuring aneurysm wall calcification directly, we expanded these groups to 150 each.

## Results

Our institutional database consisted of 984 patients with 1379 UIAs diagnosed between 2008–2021, of which 150 UIA patients were included in this study with 150 age and sex matched RIA patients (Supplementary Figure 1). Baseline characteristics are shown in Table 1. Most patients were women (196/300, 65%), and the median age was 63 years (IQR 54–68). UIA patients more often had a history of stroke, transient ischemic attack (TIA), hypertension, or hyperlipidemia compared to RIA patients, whereas RIA patients were more often current smokers. Aneurysm locations differed between groups, with UIAs being less frequently located in the anterior cerebral or communicating arteries, and more in other locations. Calcification measurements are shown in Table 2. Calcification of the aneurysm wall was found in 33/300 aneurysms (11%) and was more prevalent in UIAs (26/150, 17%) than in RIAs (7/150, 5%). This was seen in model 1 (OR 5.2, 95% CI 2.0–13.8), the conditional logistic regression model without adjustment for confounders, and it persisted after adjustment in model 2 (OR 5.8, 95% CI 1.9–17.0) and model 3 (OR 5.9, 95% CI 1.7–20.2) (Table 3). In patients with aneurysm wall calcification, the median modified Agatston score was higher in UIAs (26.7, IQR 2.5–88.4) than RIAs (10.1, IQR 1.8–14.6). For ICAC, the modified Agatston scores and prevalence were similar in UIA and RIA patients (OR 0.9, 95% CI 0.5–1.8), but UIA patients more often had severe ICAC than RIA patients (OR 2.0, 95% CI 1.1–3.6). There was no difference in the prevalence of intimal (OR 1.1, 95% CI 0.5–2.4) or medial (OR 1.0, 95% CI 0.5–2.0) locations in UIA and RIA patients. In models 2 and 3, the prevalence of ICAC, severe ICAC and ICAC location was similar between both groups. Of the covariates in models 2 and 3, aneurysms located in the anterior cerebral or anterior communicating artery were less prevalent in UIA patients, and a medical history of stroke/TIA and hyperlipidemia was more prevalent in UIA than in RIA patients (Supplementary Table 1); other covariates did not differ.

**Table 1:** Baseline patient and aneurysm characteristics

	Total (*n* = 300)	UIA (*n* = 150)	RIA (*n* = 150)
Patient characteristics			
Female sex	196 (65)	98 (65)	98 (65)
Age	63 (54–68)	63 (54–67)	63 (54–68)
Stroke/TIA	49 (16)	40 (27)	9 (6)
Hypertension^a^	140 (47)	90 (60)	50 (33)
Hyperlipidemia^a^	91 (30)	66 (44)	25 (17)
Diabetes mellitus^a^	15 (5)	7 (5)	8 (5)
Excessive alcohol intake^b^	33 (11)	17 (11)	16 (11)
Smoking status^c^			
Current	128 (43)	58 (39)	70 (47)
Former	106 (35)	58 (39)	48 (32)
Never	66 (22)	34 (23)	32 (21)
Aneurysm characteristics			
Size in mm	6.0 (4.0–9.0)	7.0 (4.3–9.8)	6.0 (4.0–8.5)
Location			
ACA/ACOM	102 (34)	31 (21)	71 (47)
ICA/PCOM	67 (22)	39 (26)	28 (19)
MCA	79 (26)	52 (35)	27 (18)
VABA	52 (17)	28 (19)	24 (16)

Values are *n* (%) or median (interquartile range)

*UIA* unruptured intracranial aneurysm, *RIA* ruptured intracranial aneurysm, *TIA* transient ischemic attack, *ACA* anterior cerebral artery, *ACOM* anterior communicating artery, *ICA* internal carotid artery, *PCOM* posterior communicating artery, *MCA* middle cerebral artery, *VABA* vertebrobasilar arteries

^a^ Data were imputed for 1 RIA patient (0.3%)

^b^ Alcohol intake was considered excessive at > 30 g per day. Data were imputed for 49 of 300 patients (16%): 14 UIA patients (9%), and 35 RIA patients (23%)

^c^ Smokers were considered former smokers if they stopped smoking > 3 months before aneurysm diagnosis. Data were imputed for 22 of 300 patients (7%): 2 UIA patients (1%), and 20 RIA patients (13%)

**Table 2:** Prevalence and severity of calcification in the aneurysm wall and intracranial carotid artery

	Total (*n* = 300)	UIA (*n* = 150)	RIA (*n* = 150)
Aneurysm wall calcification	33 (11)	26 (17)	7 (5)
Median modified aneurysm wall Agatston score (IQR)^a^	14.4 (2.3–63.0)	26.7 (2.5–88.4)	10.1 (1.8–14.6)
Median modified ICAC Agatston score (IQR)	22.4 (0.9–105.0)	26.9 (1.2–137.0)	15.8 (0.8–77.7)
Median modified ICAC Agatston score log-transformed (IQR)	3.2 (0.6–4.7)	3.3 (0.8–4.9)	2.8 (0.6–4.4)
ICAC present	249 (83)	124 (83)	125 (83)
Severe ICAC^b^	75 (25)	45 (30)	30 (20)
ICAC location			
Absent/indistinguishable	73 (24)	36 (24)	37 (25)
Intimal	88 (29)	46 (31)	42 (28)
Medial	139 (46)	68 (45)	71 (47)

Values are *n* (%) or median (interquartile range)

*ICAC* intracranial carotid artery calcification, *RIA* ruptured intracranial aneurysm, *UIA* unruptured intracranial aneurysm

^a^ For aneurysms with calcification

^b^ Severe ICAC is defined as ICAC in the fourth quartile; non-severe ICAC as first to third quartile

**Table 3:** Difference between UIA and RIA patients in aneurysm wall and intracranial carotid artery calcification

	Model 1^a^ (OR, 95% CI)	Model 2^a^ (OR, 95% CI)	Model 3^a^ (OR, 95% CI)
Aneurysm wall calcification present vs. absent	5.2 (2.0–13.8)^b^	5.8 (1.9–17.0)	5.9 (1.7–20.2)
ICAC present vs. absent	0.9 (0.5–1.8)	1.0 (0.5–2.1)	1.0 (0.4–2.2)
Severe vs. non-severe ICAC^c^	2.0 (1.1–3.6)	1.6 (0.8–3.1)	1.0 (0.4–2.3)
ICAC location			
Absent/indistinguishable	Ref.	Ref.	Ref.
Intimal	1.1 (0.5–2.4)	1.2 (0.5–3.0)	1.2 (0.4–3.4)
Medial	1.0 (0.5–2.0)	1.0 (0.5–2.2)	0.9 (0.4–2.3)

*ICAC* intracranial carotid artery calcification, *RIA* ruptured intracranial aneurysm, *UIA* unruptured intracranial aneurysm

^a^ Conditional logistic regression models. RIA patients were used as reference. All models were controlled for the matching factors age and sex. Model 1 is not adjusted for confounders. Model 2 is adjusted for aneurysm location and size. Model 3 is additionally adjusted for history of stroke or transient ischemic attack, hypertension, hyperlipidemia, diabetes mellitus, smoking status, and alcohol intake

^b^ The odds that an aneurysm with wall calcification is unruptured is 5.2 times the odds that it is ruptured

^c^ Severe ICAC is defined as ICAC in the fourth quartile; non-severe ICAC as the first to third quartile

## Discussion

Aneurysm wall calcification was more prevalent in UIA than in RIA patients, which persisted after adjustment for relevant confounders, while the prevalence of ICAC and ICAC location did not differ after adjustment.

Although our results cannot be directly compared to other studies because of heterogeneity in study populations and methods used, they seem to reinforce previous findings that calcification of intracranial aneurysms is more prevalent in UIAs than RIAs [2–4]. One study examined calcification with ex vivo micro-CT scanning in 48 UIAs and 17 RIAs, and found that 78% of all aneurysms were calcified. In UIAs this proportion was 83%, while in RIAs this was 65% [4]. The calcification fraction, the volume of calcification in the aneurysm wall relative to the total volume of the aneurysm wall, was also higher in UIAs than in RIAs [4]. Another study compared the presence of calcification on clinical CT scans in 14 UIAs to 29 RIAs in children, and found that RIAs were less often calcified (3/29, 10%) than UIAs (6/14, 43%) [2]. A third study compared the presence of calcification on clinical CT scans in 80 patients with an unruptured or ruptured giant intracranial aneurysm and similarly showed that UIAs were more often calcified (7/56, 13%) than RIAs (1/24, 4%) [3]. We demonstrated that the higher prevalence of calcification in UIA compared to RIA patients also exists in a representative adult population of UIA and RIA patients, persists after adjustment for cardiovascular risk factors, and can be observed in vivo with clinical CT scans.

We found aneurysm wall calcification in 11% of aneurysms, which is low compared to the previous studies on aneurysm wall calcification [2, 4]. The study in children found calcification in 21% of aneurysms, but 30% of the included patients had underlying pathology, such as infection or a tumor, and since calcification was more prevalent in this subgroup, this may explain the higher prevalence of aneurysm wall calcification [2]. The study using micro-CT scanning found calcification in 78% of aneurysms [4]. The resolution of micro-CT scanning is high enough to detect micro- and mesocalcifications (< 1000 µm), whereas clinical CT scanners can only detect macrocalcifications. Technical factors may thus also explain differences with our study.

Microcalcifications in atherosclerotic plaques are thought to be part of an inflammatory process that begins with macrophages infiltrating the arterial wall and releasing vesicles that initiate microcalcification [17, 18]. Microcalcifications may develop into macrocalcifications, thereby stabilizing the wall [19], but they have also been shown to concentrate in regions of damaged collagen fibers, signifying a weak arterial wall [4, 20]. We surmise that instability leads to inflammation and calcification formation, aiming to prevent rupture. If the calcification process is too slow or incomplete, rupture can still occur. Clinical photon-counting detectors have a resolution of up to 200 µm [21], and could potentially detect such microcalcifications. It would be of interest to use this technique in UIA and RIA patients, because it would help answer the question whether microcalcifications are associated with instability in intracranial aneurysms.

It seems contradictory that cardiovascular risk factors such as hypertension and older age known as risk factors for rupture [22, 23] may also protect against rupture via the buildup of calcification in the aneurysm wall [24]. The pathophysiology of calcification as a protective mechanism is not fully understood. Calcification may accumulate in response to cardiovascular risk factors, such as hypertension and smoking, but it may be too slow in some patients to prevent rupture. For example, calcification in the intimal layer may accumulate slower in older patients [4, 25].

We found a strong association between calcification in the aneurysm wall and aneurysm rupture status, while this association was not evident when using ICAC as a surrogate marker, suggesting that ICAC does not translate one-to-one to calcification in the aneurysm wall. ICAC was prevalent but calcification was less often seen in the aneurysm wall. One explanation is that ICAC may play a dual role in intracranial aneurysm rupture: on the one hand, it may represent a defense mechanism and protect against rupture; on the other hand, calcium deposition in the internal carotid artery may reduce its distensibility, thereby increasing intracranial pulse pressure, and promoting aneurysm rupture [24, 26–28]. Another explanation is that the pathophysiological processes leading to calcification in the aneurysm wall and the carotid artery may be different. Calcification in the aneurysm wall may be associated with slow, non-laminar flow [2, 29–31], whereas calcification in the internal carotid artery may be associated with high pulse pressure [32]. It has been shown by histology that UIAs contain both nonatherosclerotic and atherosclerotic calcification, whereas RIAs only contain nonatherosclerotic calcification [4], suggesting that mainly atherosclerotic calcification may be responsible for a protective effect against rupture. In the intracranial carotid artery both types are common [6] and we hypothesized that intimal calcification (predominantly atherosclerotic) is more prevalent in UIA than in RIA patients. Contrary to our hypothesis we found that ICAC location did not differ between UIA and RIA patients, which may be explained by a weak correlation between calcification location in the intracranial carotid artery and the aneurysm wall. A strength of this study is its large sample size, which enabled us to correct for confounders, unlike previous studies [2–4]. This study also has limitations. First, stroke was more prevalent among UIA compared to RIA patients, probably because we selected UIA patients based on the availability of CT imaging. In these patients the CT was often made to diagnose ischemic stroke and the UIA was an incidental finding; the UIA patients included in this study may therefore not be representative of the UIA population. A history of ischemic stroke is associated with more severe ICAC [25, 33], which could explain why aneurysm wall calcification and severe ICAC were more prevalent in our selected group of UIA patients compared to RIA patients. However, we adjusted for cardiovascular risk factors and the association between aneurysm rupture status and wall calcification persisted. Second, we may have introduced bias by collecting clinical data retrospectively. UIA patients are often in follow-up for years in the outpatient clinic, and their cardiovascular risk profile is likely to be well characterized. Because patients presenting with a RIA are admitted to the hospital in an emergency setting and do not always require outpatient follow-up, their cardiovascular risk profile is likely to be less well characterized. We may therefore have underestimated the prevalence of cardiovascular disease in the RIA group. Third, for RIA patients we had more missing data on alcohol intake and smoking status than for UIA patients, which may have limited the precision of the adjustment in model 3. Fourth, we could not present baseline data on family history, because data collection on family history was incomplete.

Our study may have clinical implications. Aneurysm wall calcification is associated with a lower risk of aneurysm rupture, but also with an increased risk of complications in neurosurgical treatment [34]. Our findings may shift the balance from elective aneurysm treatment to observation in the future, although this requires further research including risk modeling.

## Conclusions

We found that aneurysm wall calcification is more prevalent in UIA compared to RIA patients, independent of cardiovascular risk factors. We did not observe this difference when using ICAC as a surrogate marker, suggesting that calcification in the aneurysm wall does not correlate one-to-one with that in the internal carotid artery. Longitudinal studies are needed to evaluate whether the presence of calcification in the aneurysm wall is associated with a lower risk of instability and can add information about future stability to current prediction models. Because microcalcifications in the aneurysm wall probably remain undetected with clinical CT scans, ideally a more sensitive method, such as photon-counting CT, would be used to quantify them [21].

## Supplementary information

Electronic Supplementary Material

## Abbreviations

CT: Computed tomography
ICAC: Internal carotid artery calcification
RIA: Ruptured intracranial aneurysm
TIA: Transient ischemic attack
UIA: Unruptured intracranial aneurysm

## References

[1] Etminan N, de Sousa DA, Tiseo C et al (2022) European Stroke Organisation (ESO) guidelines on management of unruptured intracranial aneurysms. Eur Stroke 10.1177/23969873221099736 10.1177/23969873221099736 PMC9446328 36082246

[2] O’Brien K, Leach J, Jones B, Bissler J, Zuccarello M, Abruzzo T (2013) Calcifications associated with pediatric intracranial arterial aneurysms: incidence and correlation with pathogenetic subtypes. Childs Nerv Syst 10.1007/s00381-012-1985-4 10.1007/s00381-012-1985-4 23212467

[3] dos Santos ML, Spotti AR, dos Santos RMT et al (2013) Giant intracranial aneurysms: morphology and clinical presentation. Neurosurg Rev 10.1007/s10143-012-0407-0 10.1007/s10143-012-0407-0 22791075

[4] Gade PS, Tulamo R, Lee KW et al (2019) Calcification in human intracranial aneurysms is highly prevalent and displays both atherosclerotic and nonatherosclerotic types. Arterioscler Thromb Vasc Biol 10.1161/ATVBAHA.119.312922 10.1161/ATVBAHA.119.312922 PMC6911659 31462093

[5] Kockelkoren R, Vos A, Hecke W Van et al (2017) Computed tomographic distinction of intimal and medial calcification in the intracranial internal carotid artery. PLoS One 10.1371/journal.pone.0168360 10.1371/journal.pone.0168360 PMC5218397 28060941

[6] Vos A, Van Hecke W, Spliet WGM et al (2016) Predominance of nonatherosclerotic internal elastic lamina calcification in the intracranial internal carotid artery. Stroke 10.1161/STROKEAHA.115.011196 10.1161/STROKEAHA.115.011196 26514193

[7] Zwakenberg SR, De Jong PA, Hendriks EJ et al (2020) Intimal and medial calcification in relation to cardiovascular risk factors. PLoS One 10.1371/journal.pone.0235228 10.1371/journal.pone.0235228 PMC7357737 32658909

[8] Vos A, Kockelkoren R, de Vis JB et al (2018) Risk factors for atherosclerotic and medial arterial calcification of the intracranial internal carotid artery. Atherosclerosis 10.1016/j.atherosclerosis.2018.07.008 10.1016/j.atherosclerosis.2018.07.008 30032024

[9] Demer LL, Tintut Y (2014) Inflammatory, metabolic, and genetic mechanisms of vascular calcification. Arterioscler Thromb Vasc Biol 10.1161/ATVBAHA.113.302070 10.1161/ATVBAHA.113.302070 PMC3975044 24665125

[10] Lanzer P, Boehm M, Sorribas V et al (2014) Medial vascular calcification revisited: review and perspectives. Eur Heart J 10.1093/eurheartj/ehu163 10.1093/eurheartj/ehu163 PMC4072893 24740885

[11] Amann K (2008) Media calcification and intima calcification are distinct entities in chronic kidney disease. Clin J Am Soc Nephrol 10.2215/CJN.02120508 10.2215/CJN.02120508 18815240

[12] Janzen J, Vuong PN (2001) Arterial calcifications: morphological aspects and their pathological implications. Z Kardiol 10.1007/s003920170044 10.1007/s003920170044 11374035

[13] Li X, Du H, Yang W, Chen J, Li X, Chen X (2022) The association of renal impairment with different patterns of intracranial arterial calcification: intimal and medial calcification. Atherosclerosis 10.1016/j.atherosclerosis.2022.11.012 10.1016/j.atherosclerosis.2022.11.012 36455307

[14] Bos D, Van Der Rijk MJM, Geeraedts TEA et al (2012) Intracranial carotid artery atherosclerosis: prevalence and risk factors in the general population. Stroke 10.1161/STROKEAHA.111.648667 10.1161/STROKEAHA.111.648667 22569939

[15] Agatston AS, Janowitz WR, Hildner FJ, Zusmer NR, Viamonte M, Detrano R (1990) Quantification of coronary artery calcium using ultrafast computed tomography. J Am Coll Cardiol 10.1016/0735-1097(90)90282-T 10.1016/0735-1097(90)90282-t 2407762

[16] Pearce N (2016) Analysis of matched case-control studies. BMJ 10.1136/bmj.i969 10.1136/bmj.i969 PMC4770817 26916049

[17] New SEP, Goettsch C, Aikawa M et al (2013) Macrophage-derived matrix vesicles: an alternative novel mechanism for microcalcification in atherosclerotic plaques. Circ Res 10.1161/CIRCRESAHA.113.301036 10.1161/CIRCRESAHA.113.301036 PMC3703850 23616621

[18] Durham AL, Speer MY, Scatena M, Giachelli CM, Shanahan CM (2018) Role of smooth muscle cells in vascular calcification: implications in atherosclerosis and arterial stiffness. Cardiovasc Res 10.1093/cvr/cvy010 10.1093/cvr/cvy010 PMC5852633 29514202

[19] Rahmani R, Baranoski JF, Albuquerque FC et al (2022) Intracranial aneurysm calcification – a narrative review. Exp Neurol 10.1016/j.expneurol.2022.114052 10.1016/j.expneurol.2022.114052 PMC9058235 35346670

[20] Hutcheson JD, Goettsch C, Bertazzo S et al (2016) Genesis and growth of extracellular-vesicle-derived microcalcification in atherosclerotic plaques. Nat Mater 10.1038/nmat4519 10.1038/nmat4519 PMC4767675 26752654

[21] Van der Bie J, van Straten M, Booij R et al (2023) Photon-counting CT: review of initial clinical results. Eur J Radiol 10.1016/j.ejrad.2023.110829 10.1016/j.ejrad.2023.110829 37080060

[22] Greving JP, Wermer MJH, Brown RD et al (2014) Development of the PHASES score for prediction of risk of rupture of intracranial aneurysms: a pooled analysis of six prospective cohort studies. Lancet Neurol 10.1016/S1474-4422(13)70263-1 10.1016/S1474-4422(13)70263-1 24290159

[23] Karhunen V, Bakker MK, Ruigrok YM, Gill D, Larsson SC (2021) Modifiable risk factors for intracranial aneurysm and aneurysmal subarachnoid hemorrhage: a mendelian randomization study. J Am Heart Assoc 10.1161/JAHA.121.022277 10.1161/JAHA.121.022277 PMC8751955 34729997

[24] Bartstra JW, van den Beukel TC, Van Hecke W et al (2020) Intracranial arterial calcification: prevalence, risk factors, and consequences: JACC review topic of the week. J Am Coll Cardiol 10.1016/j.jacc.2020.07.056 10.1016/j.jacc.2020.07.056 32972537

[25] Van Den Beukel TC, Van Der Toorn JE, Vernooij MW et al (2022) Morphological subtypes of intracranial internal carotid artery arteriosclerosis and the risk of stroke. Stroke 10.1161/STROKEAHA.121.036213 10.1161/STROKEAHA.121.036213 34802249

[26] Mitchell GF (2008) Effects of central arterial aging on the structure and function of the peripheral vasculature: Implications for end-organ damage. J Appl Physiol (1985) 10.1152/japplphysiol.90549.2008 10.1152/japplphysiol.90549.2008 PMC2584844 18772322

[27] Park KY, Chung PW, Kim YB, Moon HS, Suh BC, Yoon WT (2013) Increased pulsatility index is associated with intracranial arterial calcification. Eur Neurol 10.1159/000342889 10.1159/000342889 23154455

[28] Yang H, Sayre J, Dinh H et al (2023) Image‐derived metrics quantifying hemodynamic instability predicted growth of unruptured intracranial aneurysms. Stroke Vasc Interv Neurol 10.1161/svin.122.000426 10.1161/SVIN.122.000426 PMC10118203 37090136

[29] Frösen J, Piippo A, Paetau A et al (2004) Remodeling of saccular cerebral artery aneurysm wall is associated with rupture: histological analysis of 24 unruptured and 42 ruptured cases. Stroke 10.1161/01.STR.0000140636.30204.da 10.1161/01.STR.0000140636.30204.da 15322297

[30] Choi IS, David C (2003) Giant intracranial aneurysms: development, clinical presentation and treatment. Eur J Radiol 10.1016/s0720-048x(03)00090-1 10.1016/s0720-048x(03)00090-1 12758113

[31] Cho YD, Park JC, Kwon BJ, Hee Han M (2010) Endovascular treatment of largely thrombosed saccular aneurysms: follow-up results in ten patients. Neuroradiology 10.1007/s00234-009-0622-8 10.1007/s00234-009-0622-8 19921162

[32] Melgarejo JD, Vernooij MW, Ikram MA, Zhang ZY, Bos D (2023) Intracranial carotid arteriosclerosis mediates the association between blood pressure and cerebral small vessel disease. Hypertension 10.1161/HYPERTENSIONAHA.122.20434 10.1161/HYPERTENSIONAHA.122.20434 PMC9944388 36458543

[33] Bos D, Portegies MLP, van der Lugt A et al (2014) Intracranial carotid artery atherosclerosis and the risk of stroke in whites: the Rotterdam study. JAMA Neurol 10.1001/jamaneurol.2013.6223 10.1001/jamaneurol.2013.6223 24535643

[34] Algra AM, Lindgren A, Vergouwen MDI et al (2019) Procedural clinical complications, case-fatality risks, and risk factors in endovascular and neurosurgical treatment of unruptured intracranial aneurysms: a systematic review and meta-analysis. JAMA Neurol 10.1001/jamaneurol.2018.4165 10.1001/jamaneurol.2018.4165 PMC6439725 30592482

