# Supplementary material for: Intracranial arterial calcification in patients with unruptured and ruptured intracranial aneurysms
Source: Eur Radiol. 2024 May 28;34(11):7517–25. doi: 10.1007/s00330-024-10789-2 (PMC11519314; doi:10.1007/s00330-024-10789-2)
Supplement: Supplementary file 1 — Electronic Supplementary Material [file 330_2024_10789_MOESM1_ESM.pdf]

Intracranial Arterial Calcification in Patients with Unruptured and Ruptured Intracranial Aneurysms

Electronic Supplementary Material (ESM)

Eur Radiol (2024) Kamphuis MJ, Van der Kamp LT, Lette E et al.

Supplementary Figure 1. Flowchart for selection of unruptured intracranial aneurysms

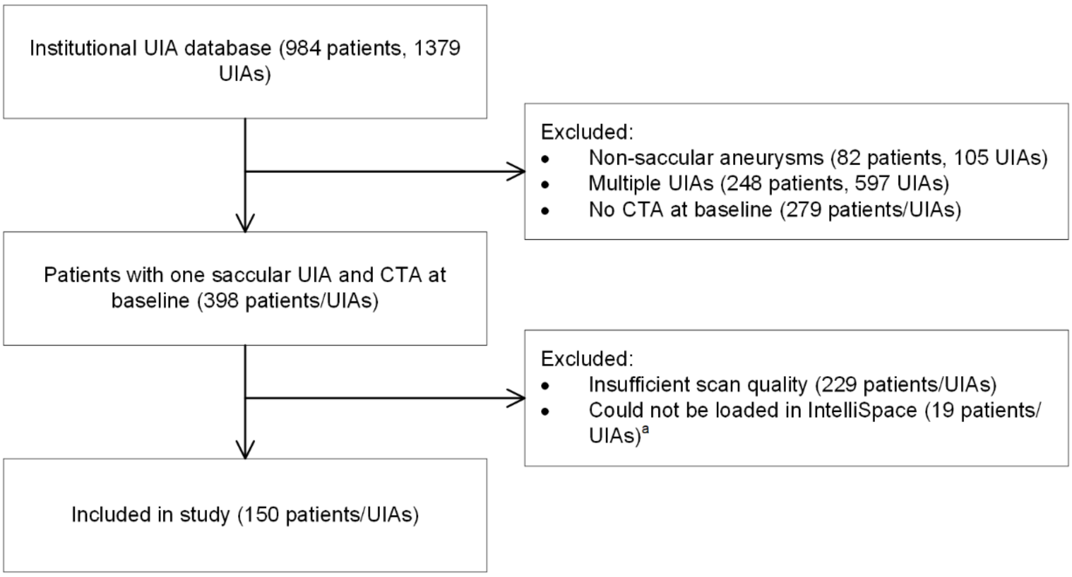

UIA, unruptured intracranial aneurysm.
[a] Nineteen patients/UIAs could not be loaded in IntelliSpace for calcification quantification, since these were saved as multiplanar reconstructions.

Supplementary Table 1. Difference between UIA and RIA patients in aneurysm wall and intracranial carotid artery calcification, with statistically significant covariates displayed

	Model 1[a] (OR, 95% CI)	Model 2[a] (OR, 95% CI)	Model 3[a] (OR, 95% CI)
Aneurysm wall calcification present vs. absent	5.2 (2.0–13.8)[b]	5.8 (1.9-17.0)	5.9 (1.7-20.2)
ACA/ACOM location	-	0.2 (0.1-0.4)	0.2 (0.1-0.5)
Stroke/TIA	-	-	3.8 (1.3-11.7)
Hyperlipidemia	-	-	2.4 (1.0-5.4)
ICAC present vs. absent	0.9 (0.5–1.8)	1.0 (0.5-2.1)	1.0 (0.4-2.2)
ACA/ACOM location	-	0.2 (0.1-0.4)	0.2 (0.1-0.5)
Stroke/TIA	-	-	3.3 (1.2-8.9)
Hyperlipidemia	-	-	2.4 (1.1-5.3)
Severe vs. non-severe ICAC[c]	2.0 (1.1-3.6)	1.6 (0.8-3.1)	1.0 (0.4-2.3)
ACA/ACOM location	-	0.2 (0.1-0.4)	0.2 (0.1-0.5)
Stroke/TIA	-	-	3.3 (1.2-9.0)
Hyperlipidemia	-	-	2.4 (1.1-5.4)
ICAC location			
Absent/indistinguishable	Ref.	Ref.	Ref.
Intimal	1.1 (0.5-2.4)	1.2 (0.5-3.0)	1.2 (0.4-3.4)
Medial	1.0 (0.5-2.0)	1.0 (0.5-2.2)	0.9 (0.4-2.3)
ACA/ACOM location	-	0.2 (0.1-0.4)	0.2 (0.1-0.5)
Stroke/TIA	-	-	3.2 (1.2-8.9)
Hyperlipidemia	-	-	2.5 (1.1-5.6)

ICAC, intracranial carotid artery calcification; RIA, ruptured intracranial aneurysm; UIA, unruptured intracranial aneurysm.

[a] Conditional logistic regression models. RIA patients were used as reference. All models were controlled for the matching factors age and sex. Model 1 is not adjusted for confounders. Model 2 is adjusted for aneurysm location and size. Model 3 is adjusted for aneurysm size, location, history of stroke or transient ischemic attack, hypertension, hyperlipidemia, diabetes mellitus, smoking status, and alcohol intake.

[b] The odds that an aneurysm with wall calcification is unruptured are 5.2 times the odds that it is ruptured.

[c] Severe ICAC is defined as ICAC in the fourth quartile; non-severe ICAC as the first to third quartile.
